# Study on feasibility of the partial meniscal allograft transplantation

**DOI:** 10.1002/ctm2.701

**Published:** 2022-01-28

**Authors:** Bao‐Shi Fan, Jing Ye, Bing‐Bing Xu, Ze‐Wen Sun, Ji‐Ying Zhang, Shi‐Tang Song, Xin‐Jie Wang, Yi‐Fan Song, Zheng‐Zheng Zhang, Dong Jiang, Jia‐Kuo Yu

**Affiliations:** ^1^ Sports Medicine Department Beijing Key Laboratory of Sports Injuries Peking University Third Hospital Beijing China; ^2^ Peking University Institute of Sports Medicine, Peking University Third Hospital, beijing, China Beijing China; ^3^ Department of Sports Medicine The Affiliated Hospital of Qingdao University Qingdao Shandong China; ^4^ Department of Orthopedics Sun Yat‐sen Memorial Hospital, Sun Yat‐sen University Guangzhou China

**Keywords:** beagle dogs, partial meniscal allograft transplantation, regeneration, total meniscal allograft transplantation, transcriptomics

## Abstract

Since the meniscus is an important stabilizing structure of the knee joint and has a significant role in load‐bearing and shock absorption, so the complete structural and functional reconstructions of the teared menisci should be done not only after partial meniscectomy but also post total meniscectomy. So far, animal experiments and good clinical practice have showed that TMAT after total meniscectomy has partially solved the problem of structural and functional reconstructions after total meniscectomy. However, partial meniscectomy will also lead to accelerated knee degeneration, and its proportion is much higher than that of patients with total meniscectomy. Herein, the feasibility of PMAT after partial meniscectomy was investigated for the first time by using the 40% posterior horn meniscectomy model of the medial meniscus in Beagle dogs, and also for the first time, TMAT group and the total meniscectomy group were used as control groups. Compared with the TMAT, the transcriptomics evaluation, scanning electron microscope observation, histological regeneration and structure, biomechanical property, inflammation environment, and the knee function post PMAT were more similar to that of normal meniscus was first reported. This study provides a PMAT scheme with clinical translational value for the complete structural and functional reconstruction of the patients with partial meniscectomy and fills the gap in the field of teared meniscus therapy on the basis of quite well clinical applications of the meniscus repair and the TMAT.

## 1 INTRODUCTION

Meniscus injury is the most common knee joint disease,^1^, ^2^, ^3^ accounting for approximately 15% of all knee athletic injuries,^4^ and is usually concomitanted with acute anterior cruciate ligament (ACL) lesions. Especially, the diagnosis rate of serious meniscus injury of the professional athletes is becoming higher and higher.^5^ Meniscus repair and total or partial meniscectomy annually were more than 1.5 million across the United States and Europe.^6^ However, the total or partial meniscectomy is inevitable for the severe meniscus tear, which has been proved as a significant inducing factor for articular cartilage degeneration and osteoarthritis,^7^, ^8^, ^9^, ^10^, ^11^ including increased ACL strain.^12^ Simultaneously, the in‐depth biomechanical basic study has recognised the irreplaceable role of the meniscus in conducting loading, dispersing stress, buffering concussion and stabilizing joints.^13^, ^14^, ^15^ Collagen meniscus implantation (CMI) began to be clinically used in 1992 as a meniscal substitute mainly for partial meniscectomy,^16^ short‐term and medium‐term clinical follow‐up have achieved satisfactory results,^17^, ^18^, ^19^, ^20^, ^21^, ^22^ but the long‐term treatment effect is difficult to guarantee.^23^ Tissue engineered meniscus (TEM) has achieved much in basic research, but the clinical translation of bionic functional TEM still has a long way to go, considering the complex heterogeneous structure of the meniscus.

Unlike CMI and TEM, the total meniscus allograft transplantation (TMAT) technique has provided an opportunity for patients with progressive joint degeneration after total meniscectomy to restore the lost meniscus and recover its function, so as to prevent the knee joint disorder with short‐, medium‐ and long‐term follow‐up.^24^, ^25^, ^26^, ^27^, ^28^, ^29^, ^30^ The TMAT technique was carried out all over the world, and finally it has been proved that it could significantly reduce joint pain, improve joint function, and prevent knee osteoarthritis (KOA). However, most of the meniscus surgery was partial meniscectomy, which also increased the stress of the cartilage and accelerate the OA progression.^10^, ^11^, ^31^ Theoretically, partial meniscus allograft transplantations (PMAT) has the superiority in smaller size and normal meniscus tissue around which was well vascularised. But there were rare reports about the experimental PMAT and even no clinical evidence on PMAT. In this study, we hypothesised that the PMAT technique can reconstruct the meniscus structure and function post‐partial meniscectomy better than the TMAT. The results of this study might recommend the PMAT technique could be used as a new procedure for the treatment of the partial meniscus defect post partial meniscectomy and provides a new option for the current meniscus injury treatment strategy.

## 2 MATERIALS AND METHODS

### 2.1 Study design

The main goal of this study was to determine whether the PMAT technique would reconstruct the normal structure and function post‐partial meniscectomy. Regenerated menisci were evaluated using systematic parameters including (i) extracellular matrix (ECM) synthesis of Glycosaminoglycans (GAGs), COL‐1, COL‐2 and COL‐3, which are primary macromolecules in the native meniscus; (ii) macroscopic and microscopic observation of the implants; (iii) biomechanical properties of the implants; (iv) regenerated meniscus transcriptome determined by RNA sequencing; and (v)macroscopic and microscopic observation of the articular cartilage.

The ability of the regenerated meniscus to delay the degeneration of articular cartilage was further investigated in vivo using 1.5–2 years old of Beagle dogs’ model with body weight of 11–15 kg. A power analysis was performed before the study as described below. Twelve weeks after the operation, 6 knees per group were collected for histological, biomechanical and transcriptome analyses. A total of 18 dogs were operated on, with all reaching the end‐points. The end‐points were predefined as the presence or the absence of significant differences in the systematic parameters listed above. All animal experiments were approved by the Animal Care and Use Committee of Peking University Third Hospital and complied with the Guide for the Care and Use of Laboratory Animals published by the National Academy Press (National Institutes of Health Publication No. 85‐23, revised 1996). Animals were euthanised before anatomy. No outliers were eliminated in the study.

### 2.2 Harvest and preparation of the allogenic menisci

Six Beagle dogs were obtained from other irrelevant studies within 6 h of their euthanasia, and their medial menisci were excised for use as the source of allografts. The dog menisci were washed in phosphate‐buffered saline (PBS, Sigma Diagnostics, St. Louis, MO) to remove residual blood and shaped into appropriate size. The allografts were then triple bagged separately, treated with 25 k Gray gamma irradiation and deeply frozen (−80°C) for transplanting.

### 2.3 Surgical procedure

Eighteen Beagle dogs were randomly allocated into part group (*n* = 6), total group (*n* = 6) and meni group (*n* = 6). Intramuscular penicillin (400 000 U) was administered preoperatively as antibiotic prophylaxis. After anaesthesia and other routine preoperative preparation, a medial parapatellar approach was made through the skin and subcutaneous tissue. A total meniscectomy was performed by resecting the right medial meniscus sharply along the periphery and detaching it from its anterior and posterior junction. The meniscus allograft was thawed in sterile saline and trimmed to a matched size. The anterior and posterior horn of the meniscus allograft were fixed in the anatomical position by means of 1.5 mm bone canal. The body of meniscus allograft was sutured to the adjacent synovium with absorbable 3‐0 sutures (Ethicon, Johnson & Johnson, Amersfoort, the Netherlands). Then, a partial meniscectomy was performed by resecting posterior 40% of the left medial meniscus but retaining the posterior horn. The size‐matched partial allograft was sutured to the recipient meniscus and the peripheral synovium tissue. For the Norm group, the operation was only performed on the medial compartment of contralateral knee joint of experimental dogs without operating on the meniscus. The joint capsule, periarticular tissues and skin were closed separately with the continuous suture. Finally, the surgical incision was isolated with sterilised gauze and the knee of dogs was externally fixed with plaster.

### 2.4 Postoperative nursing

After the operation, pain medication (non‐steroidal anti‐inflammatory drugs) and antibiotic prophylaxis were administered intramuscularly for 1 week. Dogs were in direct visual, vocal and nasal contact with each other, but were in separate pens to minimise mutual trauma. The plaster was removed 7 days and skin sutures were removed 8–10 days post‐operation. After the dogs had achieved normal gait movement with no signs of infection (2–3 weeks post‐operation), they were transferred to an IACUC‐approved farm for unrestricted movement and exercise.

### 2.5 Evaluation of knee joint function

The recovery of the experimental dogs was continuously observed after the operation and the knee joint function of each group was evaluated by Lysholm score standard at the post‐operative week 12.^32^ In this study, Beagle dogs were scored from four aspects: limp, interlocking, stairclimbing and squatting. The total score is 35.

### 2.6 Synovial fluid collection and analysis

At the end time point, synovial fluid was collected via a 1‐ml syringe with an 18‐gauge needle and assayed for IL‐1 and TNF‐α) through standard enzyme‐linked immunosorbent assay (ELISA) kits (Canine IL‐1 ELISA Kit, JM‐09526C2; Canine TNF‐α ELISA Kit, JM‐09504C2; Hermes Criterion Biotechnology).

### 2.7 Evaluation of i**mplant**s

The joints were dissected with the femur detached from the tibia and the medial meniscus remains on the tibial plateau (TP). Photographs were taken of the TP with the meniscus and the femoral condyles (FC). The menisci in each group were grossly evaluated by the Gross Evaluation of Meniscus Implant Score.^33^ The menisci and the osteochondral specimens were separated from the joints and fixed in 10% neutral buffered formalin (Sigma Diagnostics, St. Louis, MO). After fixation, the meniscus samples were cut to produce blocks that exposed the wedge‐shaped profile and showed the inner and outer zones of the regenerated meniscus in histological sections. The osteochondral specimens were then decalcified in 10% ethylenediaminetetraacetic acid (Titri‐plex III; Merck, Darmstadt, Germany) for 2 weeks. When the decalcification was completed, the osteochondral specimens were sectioned in the coronal plane at the midpoint of the TP and in the axial plane at the midpoint of the medial FC. All specimens were then dehydrated in alcohol and embedded in paraffin. Then, 5 μm‐thick sections were sliced and stained with H&E and toluidine blue (TB). The sections of menisci were treated by an immunohistochemistry procedure with the labelling of the COL‐1 and COL‐2 antibody and picrosirius red (PR) staining to distinguish COL‐1 and COL‐3. PR staining sections were analysed under polarised light. The meniscus sections were evaluated blindly according to the meniscus histology scoring system. All the sections were evaluated by three experienced researchers and the mean score was attained as the final results.

For ultrastructure observation, the menisci of the part group, total group, and normal group were processed for cryofracturing to create a cut surface in the wedge section and observed with a JSM5600LV scanning electron microscope (JEOL USA Inc.). The biomechanical properties of menisci specimens were assessed using a material testing machine (AG‐IS; Shimadzu). The samples for tensile testing were about 5 × 6 × 2 mm^3^ and the samples for compressive testing were about 4 × 3 × 2 mm^3^. The working temperature is about 22°C and the samples were kept hydrated using 0.9% NaCl solution. For the tensile testing, the samples were preconditioned to a maximum displacement of 2 mm at a displacement rate of 0.5 mm/min. The samples were tested to ultimate failure at a rate of 1 mm/min. For the compressive testing, the samples were loaded at a displacement rate of 0.5 mm/min with a maximum force of 100 N or a maximum 90% displacement. The elastic modulus was analysed from the linear portion of the stress–strain curve. Indentation assay was performed using the Tri‐boIndenter (Hysitron Inc, Minneapolis, MN, USA) with a 20 μm 90° conical probe tip to compare the surface of the regenerated and native meniscus.

### 2.8 Evaluation of joint cartilages

The cartilages of the medial FC and TP were macroscopically evaluated according to the International Cartilage Repair Society (ICRS) criteria.^34^ Three researchers who were blinded to the study scored independently and then the mean scores of the researchers were taken for the final evaluation. The paraffin section of osteochondral specimens was described above. The slides were stained with H&E and TB. Each histologic slide was scored blindly according to the Mankin scores system for cartilage degeneration.^35^

Osteochondral samples were trimmed without destroying the cartilage structure, fixed immediately in 20 ml of 25% glutaraldehyde for 24 h at 4°C, dehydrated in a graded ethanol series, and finally subjected to critical point drying for complete dehydration. The samples were coated with a 5‐nm layer of gold and then observed by JSM5600LV scanning electron microscope (SEM, JEOL, USA).

### 2.9 RNA sequencing for the meniscus transcriptome

Meniscus samples were collected from the part group, total group and norm group, respectively. We performed RNA sequencing analysis with the NovelBrain Cloud Analysis Platform. Briefly, total RNA was extracted from meniscus samples using Trizol reagent (Invi‐trogen). The cDNA libraries were then constructed for each pooled RNA sample using the VAHTSTM total RNA‐seq (H/M/R). The sequencing data were filtered with SOAPnuke (v1.5.2)^36^ by (1) removing reads containing sequencing adapter; (2) removing reads whose low‐quality base ratio (base quality less than or equal to 5) is more than 20%; (3) removing reads whose unknown base (‘N’ base) ratio is more than 5%, afterwards clean reads were obtained and stored in FASTQ format. The clean reads were mapped to the reference genome using HISAT2 (v2.0.4).^37^ Bowtie2 (v2.2.5)^38^ was applied to align the clean reads to the reference coding gene set, then the expression level of the gene was calculated by RSEM (v1.2.12).^39^ Essentially, differential expression analysis was performed using the DESeq2(v1.4.5)^40^ with *Q* value ≤ 0.05. To take an insight into the change of phenotype, Gene Ontology (GO, http://www.geneontology.org/) and Kyoto Encyclopedia of Genes and Genomes (KEGG, https://www.kegg.jp/) enrichment analysis of annotated different expressed gene were performed by Phyper (https://en.wikipedia.org/wiki/Hypergeometric_distribution) based on the hypergeometric test. The significant levels of terms and pathways were corrected by *Q* value with a rigorous threshold (*Q* value ≤ 0.05) by Bonferroni. The biological process and pathway activity networks were constructed using Cytoscape for graphical representations of enriched biological pathways with significance (*P* < 0.05), including upregulated and downregulated ones. Finally, we used the method of co‐expression analysis to focus on the gene target of meniscal regeneration in the part group. The degree and *K*‐core values of each significantly DEGs were obtained by calculating the Pearson correlation coefficient between genes. The importance of each gene for the phenotype modification was determined accordingly (the greater the degree and *K*‐core values, the greater the co‐expression ability of the indicated gene). Namely, higher ranked genes played more important roles in the whole gene network for phenotype modification than lower ranked ones. Normalised gene expression data were utilised to evaluate the relative proportions of 22 types of infiltrating immune cells via using the CIBERSORT algorithm.

### 2.10 Statistical analysis

The necessary sample size was 6 to achieve a power value of 0.8 for the parameters in this study. All statistical data were expressed as means ± SD except for Table 1 mean (range). One‐way ANOVA or two‐way ANOVA with Tukey's test was used to analyse the data. All data analyses were performed via SPSS statistical software (version 25.0, IBM‐SPSS, Armonk, NY). Values of *P* < 0.05 were considered statistically significant.

**TABLE 1:** Gross evaluation of meniscus implants

	**Part group [mean (range)]**	**Total group [mean (range)]**	***P* value**
Integration	2.8 (2–3)	2.3 (1–3)	0.209
Implant position	2.8 (2–3)	2.0 (1–3)	0.022*
Horn position	3	2.8 (2–3)	0.363
Shape	2.3 (1–3)	1.8 (1–3)	0.296
Tears	3	2.8 (2–3)	0.363
Surface	2.7 (2–3)	1.7 (1–3)	0.030*
Size	2.7 (2–3)	2.3 (1–3)	0.418
Tissue	2.7 (2–3)	1.8 (1–3)	0.049*
Synovia	2.7 (2–3)	1.7 (1–2)	0.007*
Total score	24.7 (23–26)	19.3 (16–23)	0.001*

* Statistically significant (*P* < 0.05).

## 3 RESULTS

### 3.1 Evaluation of knee joint function

From the removal of the plaster to the third week after the operation, dogs in the PMAT group (part group) and the TMAT group (total group) showed hanging hoof and weight‐free when standing, severe limp when walking, which was similar to the total meniscectomy group (meni group). In the sixth‐week post‐operation, dogs in the part group and the total group could bear the weight of the affected limb for a short time while standing and showed moderate limp when walking, but the improvement of the dogs in the meni group was not obvious. In the 12th week post‐operation, the dogs in the part group stood, walked and climbed the stairs back to normal. The dogs in the total group still had mild limp and 1 dog in this group took off both hind limbs at the same time when climbing the stairs. The dogs in the meni group were limping in different degrees, and it was slow and difficult for them to climb the stairs.

**FIGURE 1:**
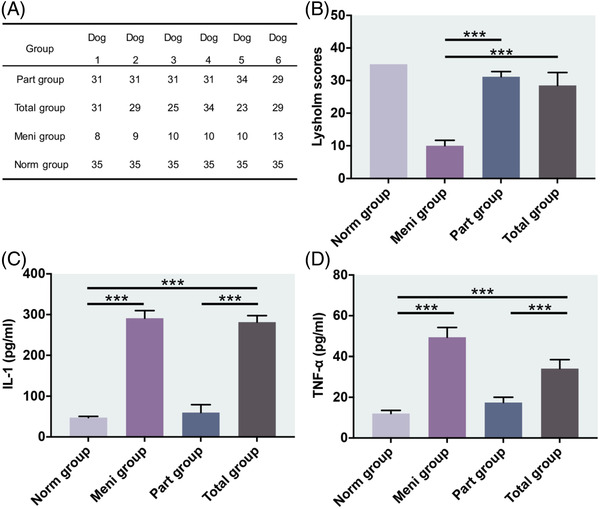
Lysholm scores of knee joint function and Synovial fluid assessment. (A,B) Lysholm scores of each group. ***P < 0.001 between the part group and meni group. ***P < 0.001 between the total group and meni group. (C,D) IL‐1 and TNF‐ α contents in knee synovial fluid collected from each study group. ***P < 0.001 between the part group and total group. All data in A, C, and D are presented as means ± SD (*n* = 6) and were analysed by one‐way ANOVA with Tukey's test

In this study, Beagle dogs were scored at week 12 postoperatively from four aspects: limp, interlocking, stairclimbing and squatting. The Lysholm score of the knee joint function (Figure 1A, B) showed that there was a significant difference between the part group and the meni group (*P* < 0.001), and there was also a significant difference between the total group and the meni group (*P* < 0.001). The scores of the part group or the total group were close to the normal control group (norm group).

### 3.2 Inflammatory factors analysis

To quantitatively analyse the inflammation of knee joint, synovial fluid was collected to analyse interleukin 1 (IL‐1; Figure 1C) and tumour necrosis factor α (TNF‐ α) (Figure 1D). Compared with the norm group, the concentration of these two inflammatory factors presented the highest level in the meni group. No obvious inflammatory reaction was observed in the part group. Compared with the part group or the norm group, the concentration of inflammatory factors in the total group was higher with a significant difference (*P* < 0.001).

### 3.3 Gross evaluation of implants

**FIGURE 2:**
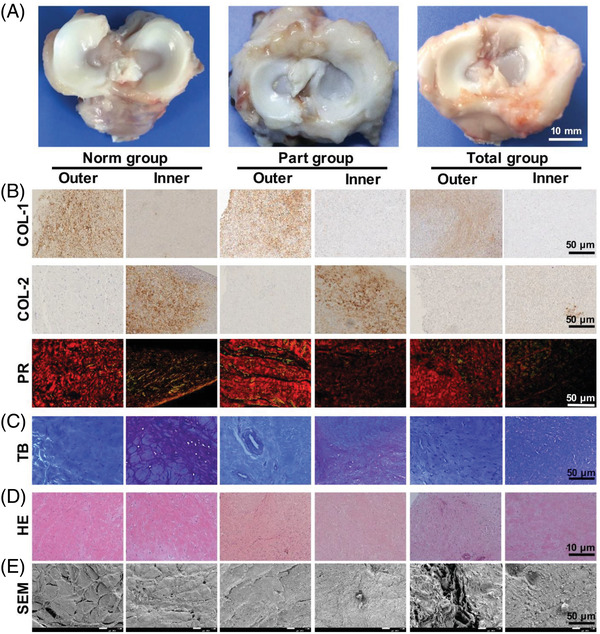
Regeneration of meniscus resembling the native tissue after 12 weeks in vivo. (A) Gross view of native or regenerated menisci at 12 weeks after in vivo implantation in Beagle dog knees. (B,C) Zone‐specific matrix phenotype analysis in regenerated versus native menisci. Tissue sections were stained by immunohistochemistry for COL‐1 and COL‐2, picrosirius red (PR) for COL‐1 and COL‐3 (B) and toluidine blue (TB) for proteoglycans (C). (D) Zone‐specific cell phenotypes in the regenerated meniscus (hematoxylin and eosin (H&E) staining). Fibroblast‐like and chondrocyte‐like cells were both observed in the implants in the part group, total group and norm group. (E) SEM images of regional variations in the ultrastructure of the implants in the part group, total group and norm group. A total of six replicates were tested, with representative images selected from the same construct

All the dogs who underwent surgery recovered well after the operation without significant weight change, infection and other complications. At postoperative 12 weeks, implants from the part group and total group were grossly evaluated (Table 1). Most of the implants healed to the normal attachment site, and there was no sign of fracture or gap formation. In the part group, the appearance of six implants was normal, white and smooth, and no obvious synovial hyperplasia or implant tears were found (Figure 2A). As shown in Table 1, the score of the total group was significantly lower than that of the part group in terms of implant position, surface, tissue and synovia. The difference of total score between the two groups was statistically significant. In the total group, two implants were slightly protruding and the synovium proliferated. The implants in the total group were soft and the surfaces were rough.

### 3.4 Histological evaluation of implants

The composition and spatial distribution of collagen in regenerated meniscus was analysed by immunohistochemistry and PR staining (Figure 2B). In PR staining, red coarse fibres represent COL‐1, while green thin fibres represent COL‐3. Compared with the total group, the part group and the norm group showed a large number of COL‐1, especially in the outer zone. Consistent with PR staining, COL‐1 immunohistochemical results showed that the staining in the total group was weaker than that in the part group. The immunohistochemical staining of COL‐2 in the part group was also stronger than that in the total group.

The norm group had strong TB heterostaining in the inner zone, and the TB heterostaining in the part group was much stronger than that in the total group (Figure 2C). According to hematoxylin and eosin (H&E) staining, the regenerated meniscus of the part group and the total group had dense cells and rich small vessels at the junction of the articular capsule and the outer edge of the implant (Figure 2D). In the part group, there were dense cells, collagen fibre formation, and good tissue healing at the junction of donor meniscus and recipient meniscus. In the total group, the number of fibrochondrocytes in the local collagen fibres of the donor meniscus decreased. Histological evaluation of the regenerated meniscus showed that there was no evidence of significant inflammatory cell infiltration or foreign body reaction in the part group, but half of the implants in the total group had foreign body reaction (Table 2). The outer zone of all regenerated menisci showed extensive vascularization and the cells were fusiform fibroblast‐like cells, and the inner zone cells were round chondrocyte‐like cells, similar to the native meniscus. In the part group, it was found that the blood vessels grew from the margin of synovium to the implant, and the cells gradually integrated into the collagen matrix of the meniscus from the synovium. Low cell areas were still observed in all implants in the total group.

**TABLE 2:** Histological features of implants

	Part group [*n* (%)]	Total group [*n* (%)]
Residual scaffold	6 (100)	6 (100)
Foreign body reaction	0	3 (50)
Hypocellular areas	4 (66.7)	6 (100)
Blood vessels	6 (100)	6 (100)
Fibrosis	3 (50)	6 (100)
Cartilage metaplasia		
Tip	6 (100)	5 (83.3)
Central	4 (66.7)	3 (50)
Integration		
Good	6 (100)	5 (83.3)
Poor	0	1 (16.7)
Inflammatory infiltrate		
Lymphocytes	1 (16.7)	2 (33.3)
Plasma	0	0
Neutrophils	0	0

*Note*. The table showed the number of implants with each of the histological features.

On the whole, the regenerated meniscus showed the zone‐specific cell phenotype. There were round chondrocyte‐like cells around the cartilage island in the inner zone, while fusiform fibroblast‐like cells in the outer zone. The zone‐specific matrix phenotype of the regenerated menisci was also similar to that of the native meniscus. The outer zone showed the arrangement of fibres dominated by COL‐1, while the inner zone showed a cartilage ECM containing COL‐2 and proteoglycan.

The results of the SEM simultaneously confirmed the zone‐specific phenotypes mentioned above. In the part group, the continuous fibrous reticular structure could be seen, the collagen fibres in the outer zone are distributed longitudinally and obliquely in many directions, and lots of round cells with cell protuberances were around cartilage lacuna in the inner zone, which was similar to that of the norm group (Figure 2E). The collagen fibres in the total group were loosely distributed in areas with few cells.

### 3.5 Biomechanical evaluation of implants

**FIGURE 3:**
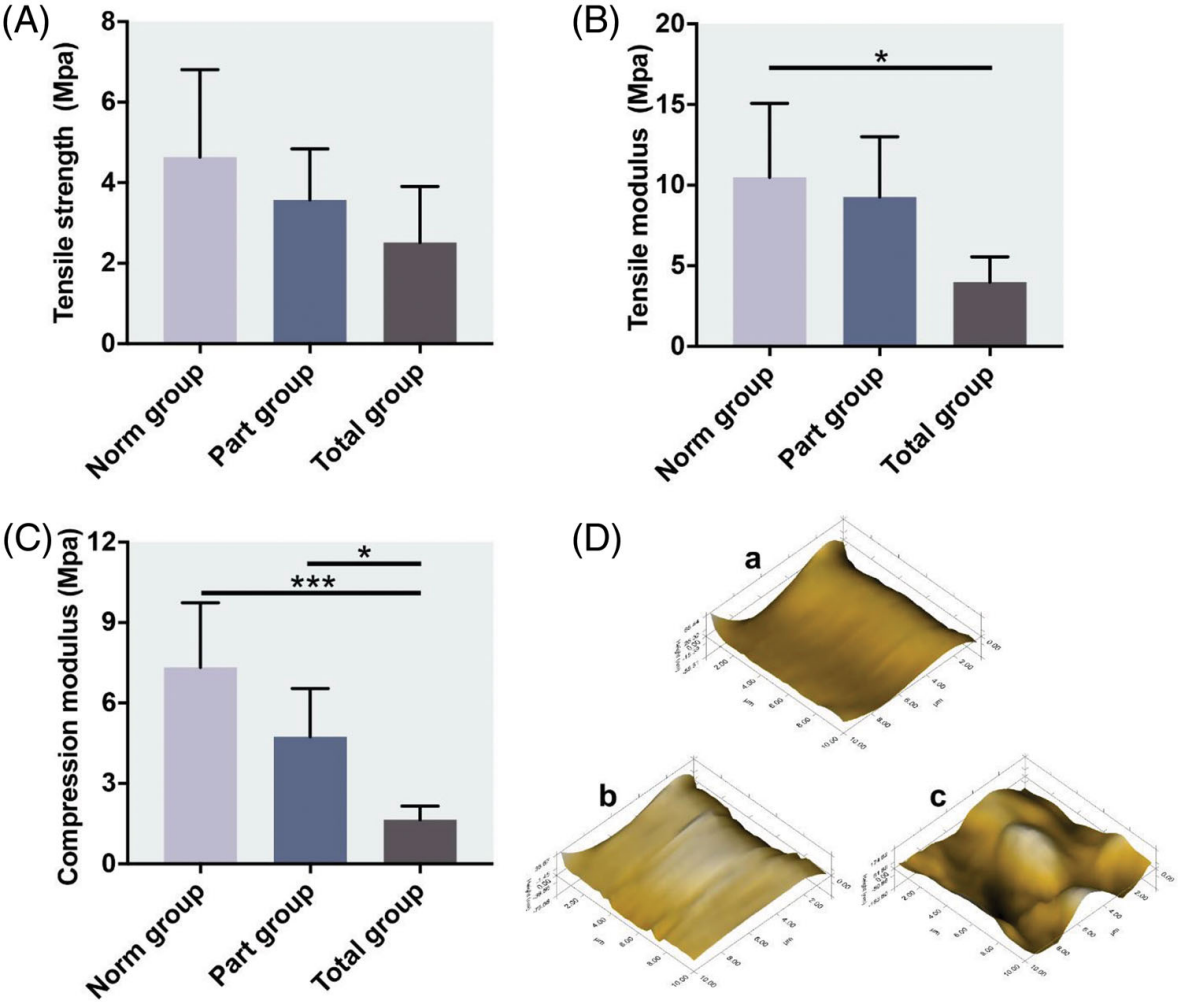
Mechanical properties of regenerated meniscus after 12 weeks in vivo. (A) Tensile strength. (B) Tensile modulus. *P < 0.05 between the norm group and total group. (C) Compression modulus. *P < 0.05 between the part group and total group. ***P < 0.001 between the norm group and total group. (D) Microscopic geomorphology of the femoral condylar contact surface of menisci from norm group (a), part group (b) and total group (c) was acquired during nanoindentation. All data in A, B and C are means ± SD and were analysed by one‐way ANOVA with Tukey's test

To further evaluate the quality of the implants, we tested the biomechanical properties of the implants, including ultimate tensile strength, tensile modulus and compression modulus. In the tensile test, the implants in the part group and the total group recovered the ultimate tensile strength similar to the native meniscus, especially the part group was closer to the norm group (Figure 3A). The tensile modulus of the total group was significantly lower than the norm group (*P *< 0.05). And the tensile modulus of the part group was higher than that of the total group (Figure 3B). In the compression test, although the values in the norm group were higher than those in both part group and the total group, the difference between the part group and the norm group was not statistically significant (Figure 3C). The compression modulus of the part group was significantly higher than the total group (*P *< 0.05) which was significantly lower than the norm group (*P *< 0.001). According to microscopic geomorphology, the surfaces of implant in the part group (Figure 3D, b) were as smooth as native meniscus (Figure 3D, a), but in the total group (Figure 3D, c) the surfaces were uneven.

### 3.6 Analysis of meniscus transcriptome by RNA sequencing

**FIGURE 4:**
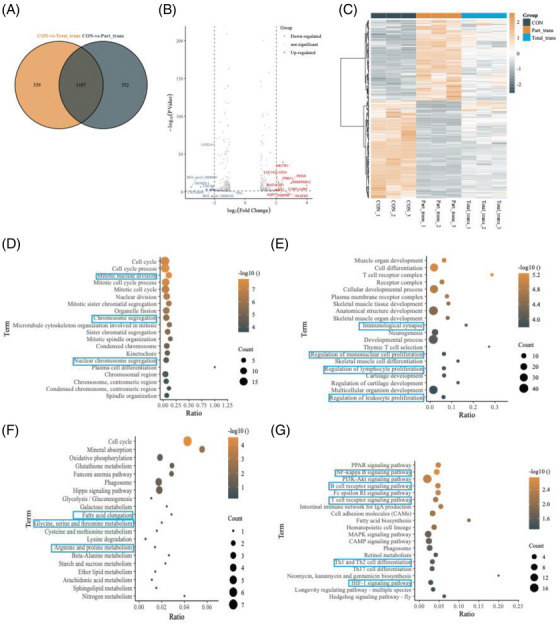
Meniscus transcriptome determined by RNA sequencing. (A) 352 DEGs outside the intersection in part group were acquired from Venn analysis. (B) Volcano plots analysis for differentially expressed genes (DEGs). (C) Heat map analysis for the repeatability of intra‐group samples and the differences in gene expression between groups. (D,E) Gene ontology (GO) analysis for biological process, D (UP‐gene), E (DOWN‐gene). (F,G) Pathway analysis for different express genes (DEGs), F (UP‐gene), G (DOWN‐gene)

To analyse the gene targets and potential signal pathways of better meniscal regeneration phenotype in the part group, we performed RNA sequencing in the meniscal specimens from the part group, total group and norm group, respectively. The Venn map (Figure 4A) and Volcano plot (Figure 4B) showed that there were 352 differentially expressed genes (DEGs) in the part group outside the intersection with the total group, which may be the gene targets with better tissue phenotype in the part group. The heat map (Figure 4C) obtained by cluster analysis of the 352 DEGs showed that the repeatability of intra‐group samples was good and there were differences in gene expression between groups. The GO analysis and KEGG pathway analysis revealed the biological function and pathway changes of these DEGs. The GO analysis showed that mitotic nuclear division, chromosome segregation and nuclear chromosome segregation were upregulated (Figure 4D), while biological processes including immunological synapse, regulation of mononuclear cell proliferation, regulation of lymphocyte proliferation and regulation of leukocyte proliferation were downregulated (Figure 4E). The KEGG pathway analysis indicated that the Hippo signalling pathway, glycolysis/glucoreogenesis and glycine, serine and threonine metabolism were significantly upregulated (Figure 4F). On the contrary, NF‐kappa B signalling pathway, B cell receptor signalling pathway, T cell receptor signalling pathway, Th1 and Th2 cell differentiation and HIF‐1 signalling pathway were downregulated (Figure 4G). MCODE calculations were used to seek core regulatory genes involved in the part group.

**FIGURE 5:**
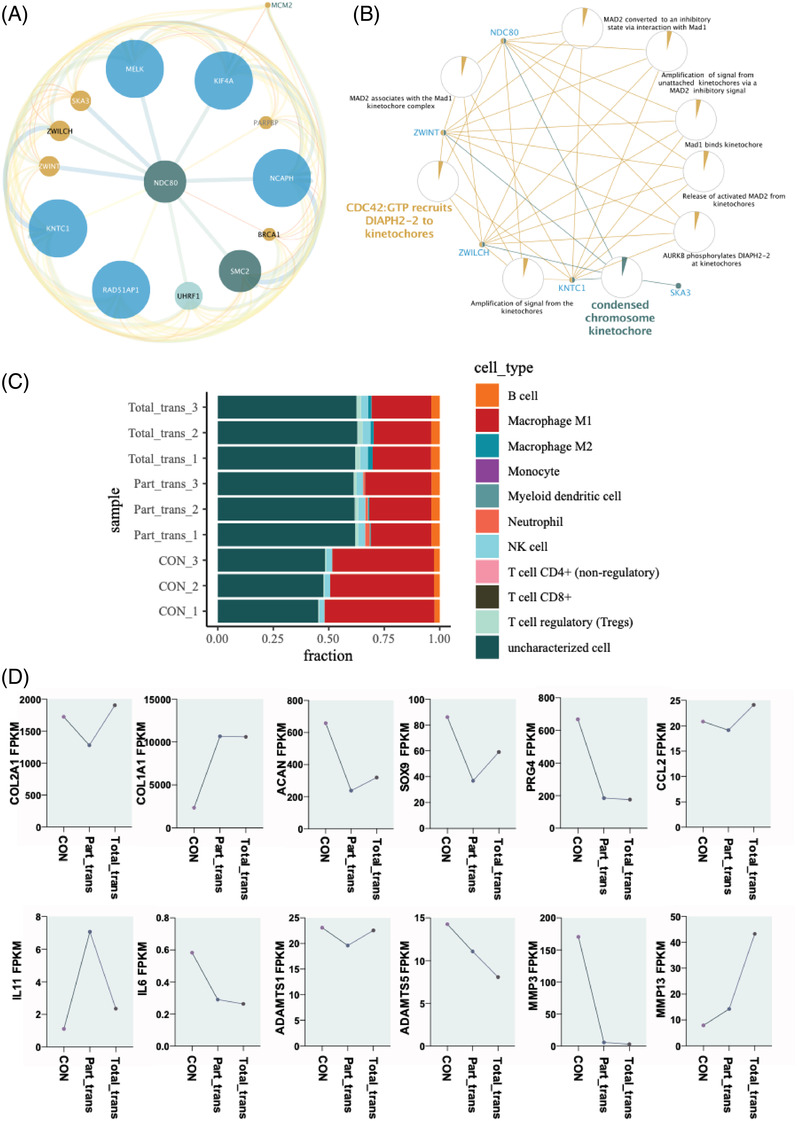
Interaction network and immune infiltration analysis. (A) The most important gene clusters of DEGs. (B) GO, KEGG and KEY GENE interaction network of the first gene cluster. (C) Immune infiltration analysis of the first gene cluster. (D) Maker gene analysis of three indicated groups

We found that these 14 genes (*KNTC1*, *RAD51AP1*, *UHRF1*, *SMC2*, *BRCA1*, *NCAPH, PARPBP, KIF4A, MELK, SKA3, ZWILCH, ZWINT and MCM2*) were at the top of the cluster and most likely to be related to the structural and functional regeneration of the meniscus (Figure 5A). Tissue regeneration activities were significantly enriched according biological process network of GO and KEGG analysis (Figure 5B). In order to further reveal the immune microenvironment in regeneration of the meniscus, the CIBERSORT method was used to analyse specific immune cell types that infiltrated into meniscus tissue. Among the 22 types of immune cells investigated, the results showed that macrophage levels were significantly lower in regeneration meniscus, Further, the satisfactory phenotype of the partial meniscal graft group relative to the total meniscal graft group may be due to a specific degree of immune infiltration including fewer T cell regulatory, fewer NK cells, more neutrophil cells, more M1 cells, and fewer M2 cells (*p* < 0.05) (Figure 5C). Further, we observed a number of maker genes at the transcriptional level in the meniscal chondrocytes of the partial meniscal transplantation group including relative suppression of COL2A1, high activation of COL1A1, and relative low expression of ACAN, SOX9, and PRG4, as well as moderate suppression of ECM protein maker genes including ADAMTS1, ADAMTS5 and MMP3, and moderate activation of MMP13. We also found significant activation of inflammatory genes including IL‐11 and relative suppression of IL‐6, which might be the reason why some meniscus transplantation groups recovered phenotypes close to normal groups (Figure 5D).

### 3.7 Chondroprotection evaluation

**FIGURE 6:**
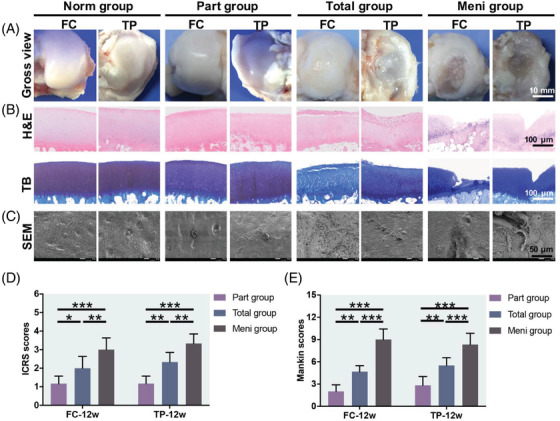
Gross and microscopic observations of cartilage 12 weeks after implantation. (A) Gross view of the femoral condyle (FC) and tibial plateau (TP). (B) H&E and TB staining of articular cartilage in the FC and TP. (C) SEM images of articular cartilage in the FC and TP. (D) ICRS and (E) Mankin scores assessing cartilage degeneration. *P < 0.05, **P < 0.01 and *P* < 0.001 between the indicated groups. All data are means ± SD (*n* = 6) and were analysed by two‐way ANOVA with Tukey's test

According to the macroscopic evaluation of articular cartilage, no obvious tear was found in the part group. In the total group, the surface of cartilage lost luster, being rough and accompanied with varying degrees of damage. In the meni group, the cartilage was seriously damaged, and even the subchondral bone could be seen with the naked eye (Figure 6A). H&E and TB staining (Figure 6B) of the FC and the TP in the meni group showed that the cartilage structure was destroyed, the boundary between calcified layer and bone tissue was blurred, the number of chondrocytes decreased greatly, the local tidal line was broken and the heterostaining degree of TB decreased heavily. In the total group, cracks appeared in the surface layer of cartilage, chondrocytes were unevenly distributed, the heterostaining degree of TB decreased moderately, and irregular changes could be seen on the surface of FC and TP. In the part group, the boundary line of the whole layer structure of cartilage was clear, the tidal line was intact, the number of cells in each layer was normal, the nucleus was deeply stained and TB heterostaining was obvious, which was similar to that in the norm group. According to the ICRS and Mankin scores, cartilage wear was the most serious in the meni group. The articular cartilage score of FC and TP in the part group and total group were significantly lower than those in the meni group. ICRS (Figure 6D) and Mankin scores (Figure 6E) in the total group were significantly higher than those in the part group.

The microscopic changes of articular cartilage were further observed by the SEM (Figure 6C). In the norm group, the surface of FC and TP is smooth, and a large number of chondrocytes can be seen. The cartilage surface of the part group was not cracked, the structure of each layer was intact, and the number of cells was similar to that of the norm group. On the contrary, the cartilage surface of the total group was cracked, the structure was destroyed and the number of cells decreased. The cartilage surface of the meni group had deep fissures wrapped in worn fibres.

## 4 DISCUSSION

In this study, our findings showed that the partial meniscus transplantation could successfully reconstruct the meniscal histological structure and biomechanics well, could protect cartilage in an inflammation environment enon par with those of the norm group. The PMAT technique showed the superiority in the tissue regeneration, biomechanical characteristics, inflammation stimulating and the chondroprotection compared with the TMAT technique.

The part group and the total group showed not only reconstruction of the histological structure of the meniscus in terms of cellular and ECM level but also maintenance of the biomechanical properties of the knee joint. The Lysholm score indicated that the MATs’ techniques could restore normal knee joint function after total and partial meniscectomies in dogs. Both of the ICRS and Mankin scores showed the chondroprotective effect of both MATs’ techniques, especially no obvious cartilage degradation was seen in the part group. The results might be related to the histological and biomechanical remodelling of implant which played an important role in the postoperative procedures. The histological reconstruction of implant is the basis of the biomechanical function. In theory, the meniscus tissue has zone‐specific GAGs and collagens that are responsible for the compressive and tensile loading from relative motion within the joint.^14^, ^41^ The COL‐2 and GAGs expressed in the inner zone of the meniscus carry the compressive loading, which is usually an important factor in cartilage differentiation,^42^, ^43^ while the COL‐1 expressed in the outer zone carries the tensile loading increasing fibrogenesis,^44^, ^45^ increased tensile strength. Tensile modulus and compressive modulus of the implants from the part group and the total group enabled joint loading distribution on par with that of the norm group. Our previous studies have shown that almost all of the fibrochondrocytes of implant appeared necrosis after irradiation and deep cryopreservation, and the implant is equivalent to an acellular scaffold.^46^, ^47^ These enhanced biomechanical properties above may be attributed to the ingrowth of autologous tissue cells and they differentiated into fibrochondrocytes that can secrete ECM.^15^, ^48^ Compared with simple meniscectomy, the cartilage evaluation results proved that the MAT technique could effectively delay the speed of cartilage degeneration.

Interestingly, the therapeutic effect of the PMAT technique for partial meniscectomy was better than the TMAT technique. Compared with the total group, the compressive modulus was higher in the part group, probably through more deposition of COL‐2 and GAGs in the inner zone. On the other hand, the expression of COL‐2 and GAGs of the total group in the inner zone was relatively less, and the ability to carry compressive loading was weak, which led to more mechanical loading on cartilage and accelerated joint degeneration. Histology and ultrastructure observation of implants showed that the number of fibrochondrocytes in the part group was more than that in the total group, and our transcriptome results also proved that a large number of genes and biological processes related to cell division and proliferation were significantly up‐regulated in the part group. Besides, the analysis of inflammatory factors in synovial fluid showed that the inflammatory response in the part group was not obvious, and the evidence of inhibition of inflammatory response and immune response signal pathways was also found by the transcriptome analysis. Therefore, the PMAT technique retained as much native meniscus tissue as possible, and reconstructed the normal histological structure and biomechanical properties of the meniscus without causing obvious inflammatory reaction and immune response, protecting cartilage and the knee joint function. We speculate that the PMAT technique will also achieve satisfactory results for partial meniscus injuries.^49^

Our previous researches have confirmed that the TMAT technique could be a promising alternative for total meniscectomy in rabbit models with tissue regeneration and the ability to protect cartilage.^46^, ^47^ Our short‐term clinical follow‐up results also proved that the TMAT technology can relieve joint pain, swelling and other symptoms, promote the recovery of the joint function and improve the stability of the knee joint.^50^ For the great majority of patients, many authors have reported the short‐, medium‐ and long‐term satisfactory clinical outcomes following TMAT, but long‐term follow up results showed quite low survivorship of the transplanted meniscus.^9^, ^26^, ^27^, ^28^, ^29^, ^30^ This study using a dog model not only verified the effectiveness of the TMAT technique on total meniscectomy but also obtained the result that the PMAT technique can reconstruct the structure and function of the meniscus better after partial meniscectomy. Unlike our study, Strauss et al.^49^ performed the segmental meniscal allograft transplantation within the white zone of the central medial meniscus defect of six sheep with fresh‐frozen allografts. Partial meniscectomy within this central white zone usually means little tissue loss compared with most of the partial meniscectomies within the posterior horn, which often lost 25%–30% meniscus tissue of the medial meniscus, and could cause more than 50%– 70% meniscectomy if it extends to the middle part of the meniscus. Strauss et al.^49^ reported partial healing of the segmental meniscal allograft to the remnant meniscal tissue by displaying a semi‐quantitative histological analysis of the implants at 90 days. However, in addition to setting up the meni group and the total group as two control groups, our study also had a comprehensive evaluation system, including postoperative knee joint function evaluation, inflammatory factor analysis, histological and ultrastructural observation, biomechanical properties comparison and potential gene target mining behind regeneration phenotype. Nyland et al. using a porcine model in vitro evaluated the tibiofemoral contact pressures of partial medial meniscal grafting for the defect of the central 1/3 of the meniscus and successfully normalised medial tibiofemoral joint compartment pressure magnitudes, areas and locations relative to a native meniscus at different knee flexion angles.^51^

The qualifying criteria for meniscal regeneration include the reconstruction of the histological structure and the recovery of the biomechanical function.^52^, ^53^ Few studies on the application of the PMAT technique in partial meniscectomy were reported. No previous literature has shown multi‐evaluations of histology, biomechanics, inflammation, cartilage protection and transcriptomics of the regenerated meniscus after PMAT compared with the meni group and the TMAT group, though some studies have shown separate histological analysis^49^ or pressure measurement.^51^ We applied the MAT techniques to repair different meniscal defects using a dog model, which not only verified that the MAT technique could reconstruct the structure and function post‐partial and total meniscectomy but also found that the repair effect of the PMAT technique was better than that of the TMAT technique. Finally, we excavated the possible gene targets, activated signalling pathways and cellular functions behind this superiority, which can be used as the basis for future researches. Data mining of the transcriptome of the meniscal regeneration region also suggested us a lot of useful information: unique alterations in the degree of immune infiltration in the meniscal partial graft group, partial suppression of chondrocyte transcription factor levels in the regeneration region of the meniscal partial graft group, inflammatory factor infiltration, and activation of MMP13 are important potential tissue repair mechanisms, which are more important directions for our further research on how to promote repair after meniscal injury at later stage. We speculate that the treatment strategy of partial meniscus injuries which cannot be sutured surgically will shift from the total transplantation to partial transplantation with allogeneic meniscus transplantation, and suit for the bionic biological scaffold implantation much better compared with the TMAT.

The major limitation of this study was that we did not carry on the experimental verification to the screened gene targets. Although accurate targeting of the molecular mechanism related to meniscal regeneration is challenging, future studies using advanced bioinformatics analysis and gene manipulation methods will certainly find closely related molecules or signalling pathway which can promote meniscal regeneration and reduce osteoarthritis severity, such as transcription factors.^1^ Meanwhile, we did not research the cell source of the regenerated meniscus. In particular, the cells of the regenerated meniscus tissue in the part group may come from the synovial tissue or from the residual meniscus. Second, our animal experiments were evaluated after 12 weeks and the observation duration was relatively short. However, the results showed a good trend and the long‐term healing effect of the PMAT technique in repairing partial meniscectomy needs to be further studied. Furthermore, we chose the back 40% of the meniscus as the repair position in this study which is the most common clinical strategy of partial meniscectomy. If necessary, the study repairing meniscal defects with the PMAT technique in different locations and areas can be put on the agenda. For clinical translation, studies on a large sample from either animal experiments or clinical trials are essential.

To establish a reasonable treatment strategy for the refractory meniscus injuries, we evaluated the regenerated meniscus after partial and total meniscal allograft transplantation. The implants in the part group and the total group resumed the histological structure and biomechanical properties similar to those of native menisci, and we also found that the PMAT technique could be an alternative strategy for partial meniscectomy. Therefore, we propose a new surgical method to repair partial meniscectomy, PMAT technique. This PMAT technique verified by a large animal model could provide a reliable basis for the clinical translation. Our study results may provide a comprehensive repair strategy for the treatment of clinical refractory meniscus injuries. The surgical suture can be performed on meniscal injuries allowed to be sutured, such as a simple local tear in the red‐area of meniscus, the PMAT technique can be used for those who underwent partial meniscectomy, and the TMAT technique can only be used for those who have no choice but to undergo total meniscectomy. At present, the researches on tissue‐engineered meniscus transplantation are in full swing, and they have achieved good results in the treatment of meniscus injury in animals, but there is still a long way to go before clinical translation. Furthermore, our research idea provides the consideration for the application in PMAT of the tissue‐engineered meniscus.

## 5 CONCLUSIONS

In this study, we demonstrated that the PMAT technique can reconstruct the normal histological structure and the biomechanical function post‐partial meniscectomy and achieve a chondroprotective goal. This strategy provides a comprehensive treatment strategy for clinical refractory meniscus injuries, especially the PMAT technique can be the choice after partial meniscectomy. In the near future, for the menisci injuries, if it could be sutured and repaired, suture and repair should be done, the PMAT could be done for meniscus tear which was irreparable, and the TMAT for total meniscectomy. In this way, we can achieve the ideal of reconstructing the structure and function of the injured meniscus for patients suffered from any kind of meniscus tears.

## CONFLICT OF INTERESTS

The authors declare that there is no conflict of interest that could be perceived as prejudicing the impartiality of the research reported.
